# Supplementary material for: Impact of Ferulated Arabinoxylans from Maize Bran on Farinograph and Pasting Properties of Wheat Flour Blends
Source: Foods. 2024 Oct 26;13(21):3414. doi: 10.3390/foods13213414 (PMC11545305; doi:10.3390/foods13213414)
Supplement: Supplementary file 1 [file foods-13-03414-s001.zip › Supplementary File 1.pdf]

Supplementary File 1

Figure 1; Farinograms T0 to T5 (duplicate samples)

Brabender® Farinograph

Sample: T0 Control Rep 1
Date: 7/1/2024 6:44:55 PM

Method: AACC
Operator: TENG

Mixer: 50 g Speed: 63 1/min
Consistency 503 FU with waterabsorption 54.8 %

Moisture content: 15.6 %

Waterabsorption (corrected for 500 FU):	54.9	%
Waterabsorption (corrected to 14.0 %):	56.8	%
Development time:	7.8	min
Stability:	14.6	min
Toleranceindex (MTI):	28	FU
Time to breakdown:	13.3	min
Farinograph quality number:	133	
Remarks:		

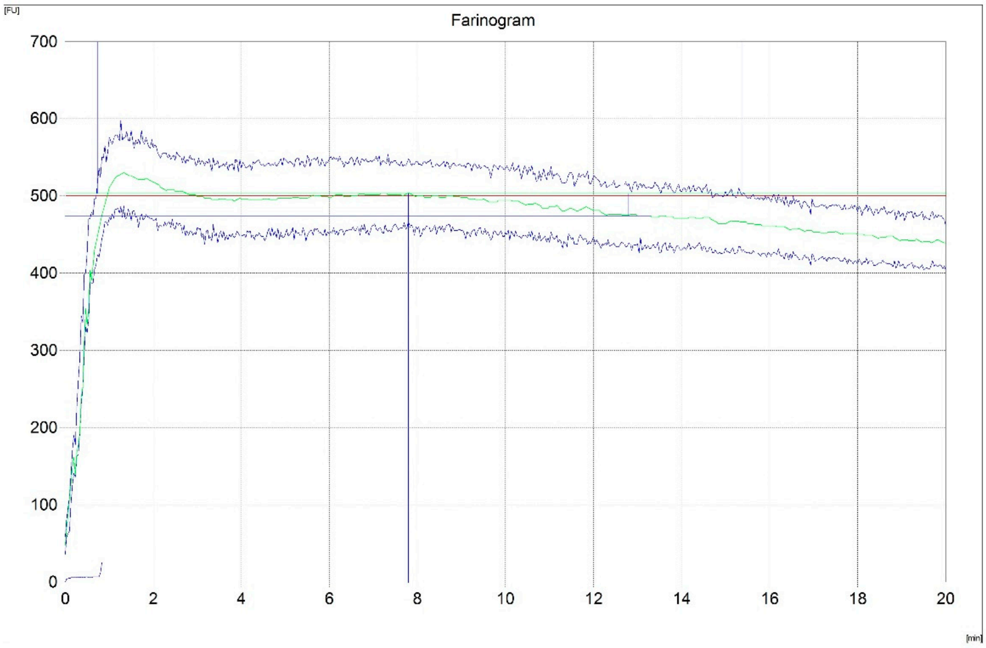

Test: C:\Users\cwbrabender\Documents\End Users 2024\T0 Control Rep 1.FAD

Brabender® Farinograph

Sample: T0 Control Rep 2
Date: 7/2/2024 3:56:10 PM

Method: AACC
Operator: TENG

Mixer: 50 g   Speed: 63 1/min
Consistency 499 FU with waterabsorption 54.9 %

Moisture content: 15.6 %

Waterabsorption (corrected for 500 FU):	54.9	%
Waterabsorption (corrected to 14.0 %):	56.8	%
Development time:	7.7	min
Stability:	14.0	min
Toleranceindex (MTI):	26	FU
Time to breakdown:	13.1	min
Farinograph quality number:	131	
Remarks:		

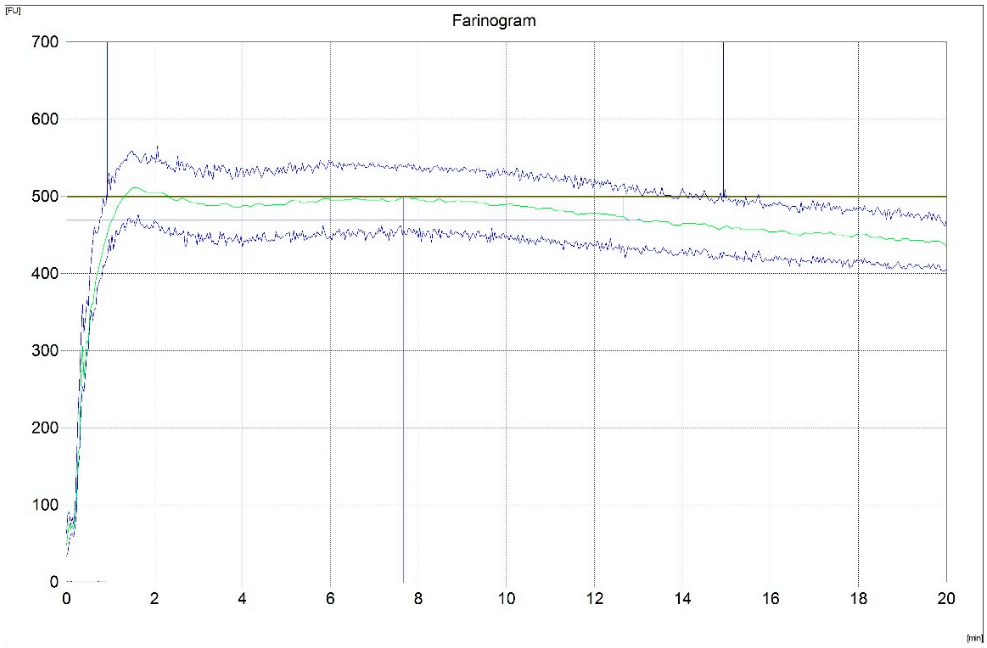

Test: C:\Users\cwbrabender\Documents\End Users 2024\T0 Control Rep 2.FAD

Brabender® Farinograph

Sample: T1Rep 1
Date: 7/1/2024 7:15:21 PM

Method: AACC
Operator: TENG

Mixer: 50 g Speed: 63 1/min
Consistency 524 FU with waterabsorption 54.8 %

Moisture content: 15.7 %

Waterabsorption (corrected for 500 FU):	55.4	%
Waterabsorption (corrected to 14.0 %):	57.4	%
Development time:	24.2	min
Stability:	40.2	min
Toleranceindex (MTI):	6	FU
Time to breakdown:	44.3	min
Farinograph quality number:	443	
Remarks:		

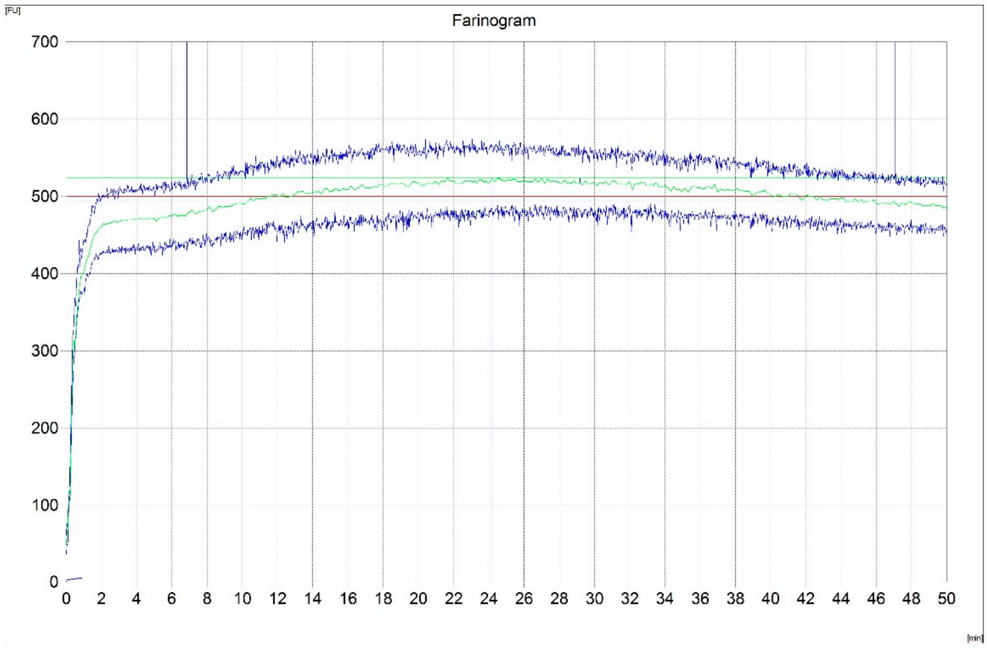

Test: C:\Users\cwbrabender\Documents\End Users 2024\T1 REP 1.FAD

Brabender® Farinograph

Sample: T1 Rep 2
Date: 7/2/2024 5:04:52 PM

Method: AACC
Operator: TENG

Mixer: 50 g Speed: 63 1/min
Consistency 511 FU with waterabsorption 56.6 %

Moisture content: 15.7 %

Waterabsorption (corrected for 500 FU):	56.9	%
Waterabsorption (corrected to 14.0 %):	58.9	%
Development time:	25.2	min
Stability:	43.4	min
Toleranceindex (MTI):	5	FU
Time to breakdown:	48.6	min
Farinograph quality number:	486	

Remarks:

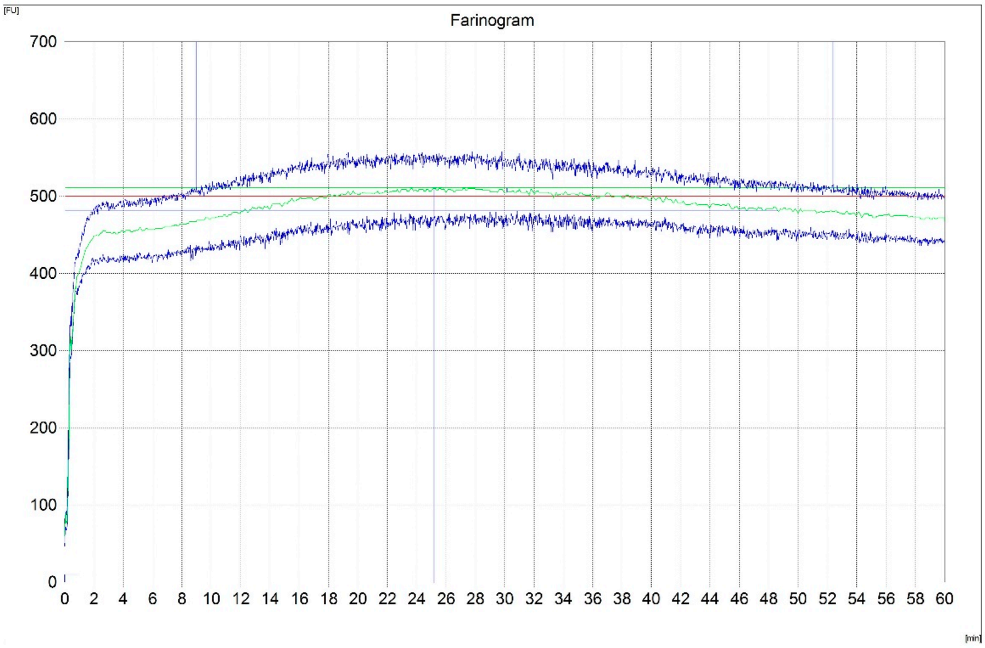

Test: C:\Users\cwbrabender\Documents\End Users 2024\T1 Rep 2.FAD

BRABENDER® 2.3.0

Brabender® Farinograph

Sample: T2 Rep 1
Date: 7/1/2024 9:46:29 PM

Method: AACC
Operator: TENG

Mixer: 50 g Speed: 63 1/min
Consistency 483 FU with waterabsorption 57.8 %

Moisture content: 15.5 %

Waterabsorption (corrected for 500 FU):	57.4	%
Waterabsorption (corrected to 14.0 %):	59.2	%
Development time:	3.3	min
Stability:	5.0	min
Toleranceindex (MTI):	54	FU
Time to breakdown:	6.6	min
Farinograph quality number:	66	

Remarks:

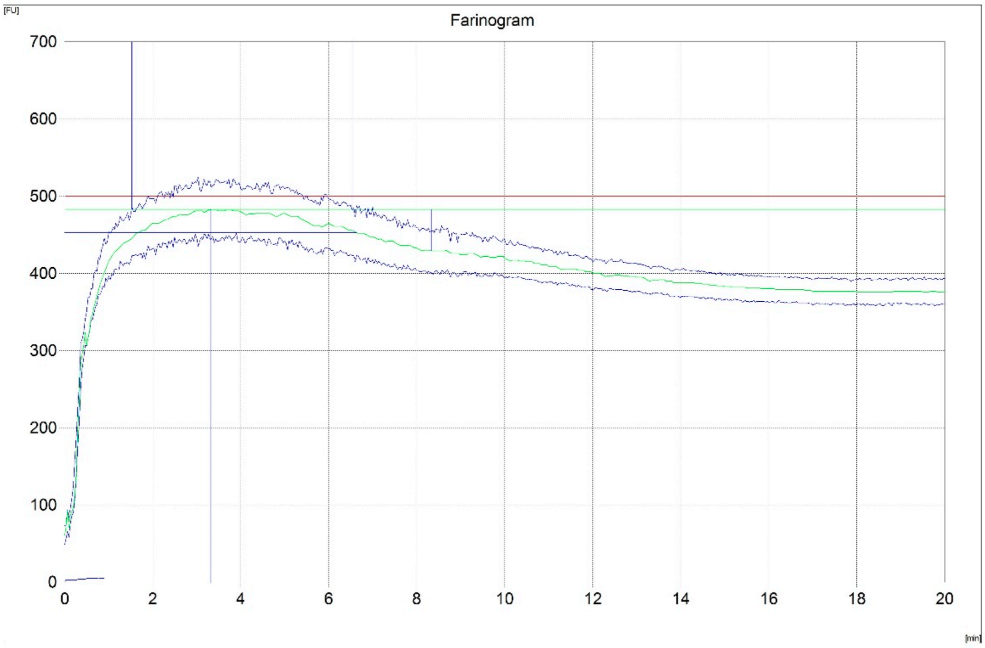

Test: C:\Users\cwbrabender\Documents\End Users 2024\T2 Rep 1.FAD

Brabender® Farinograph

Sample: T2 Rep 2 | Method: AACC
Date: 7/2/2024 6:15:54 PM | Operator: TENG

Mixer: 50 g   Speed: 63 1/min | Moisture content: 15.5 %
Consistency 486 FU with waterabsorption 57.4 %

Waterabsorption (corrected for 500 FU):	57.1	%
Waterabsorption (corrected to 14.0 %):	58.9	%
Development time:	4.0	min
Stability:	5.4	min
Toleranceindex (MTI):	57	FU
Time to breakdown:	6.4	min
Farinograph quality number:	64	
Remarks:		

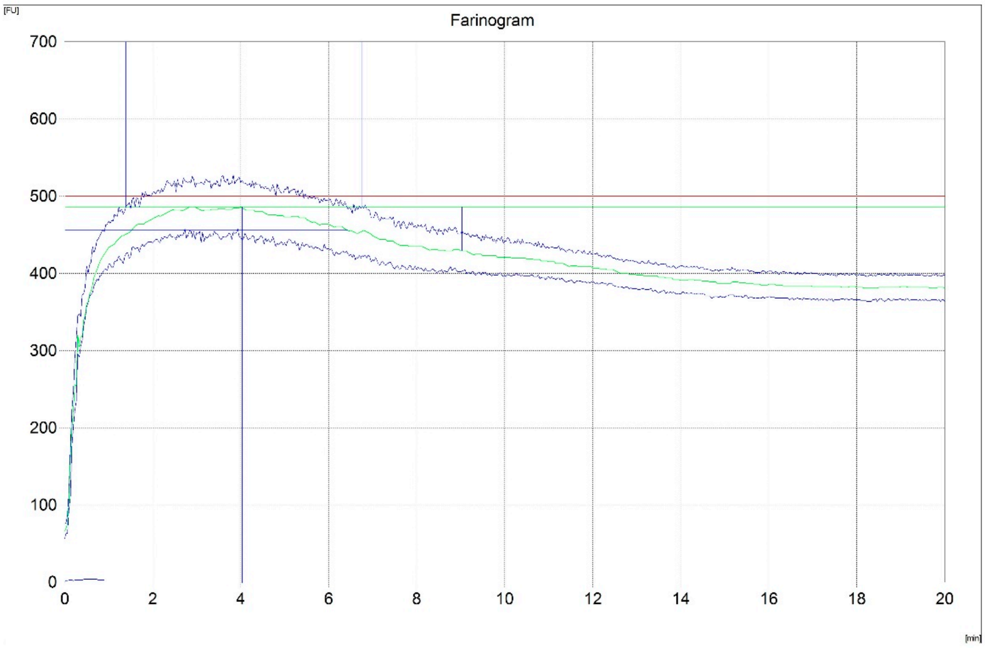

Test: C:\Users\cwbrabender\Documents\End Users 2024\T2 Rep 2.FAD

Brabender® Farinograph

Sample: T3 Rep 1
Date: 7/1/2024 10:14:00 PM

Method: AACC
Operator: TENG

Mixer: 50 g Speed: 63 1/min
Consistency 479 FU with waterabsorption 58.0 %

Moisture content: 15.8 %

Waterabsorption (corrected for 500 FU):	57.5	%
Waterabsorption (corrected to 14.0 %):	59.6	%
Development time:	2.8	min
Stability:	4.1	min
Toleranceindex (MTI):	58	FU
Time to breakdown:	5.4	min
Farinograph quality number:	54	

Remarks:

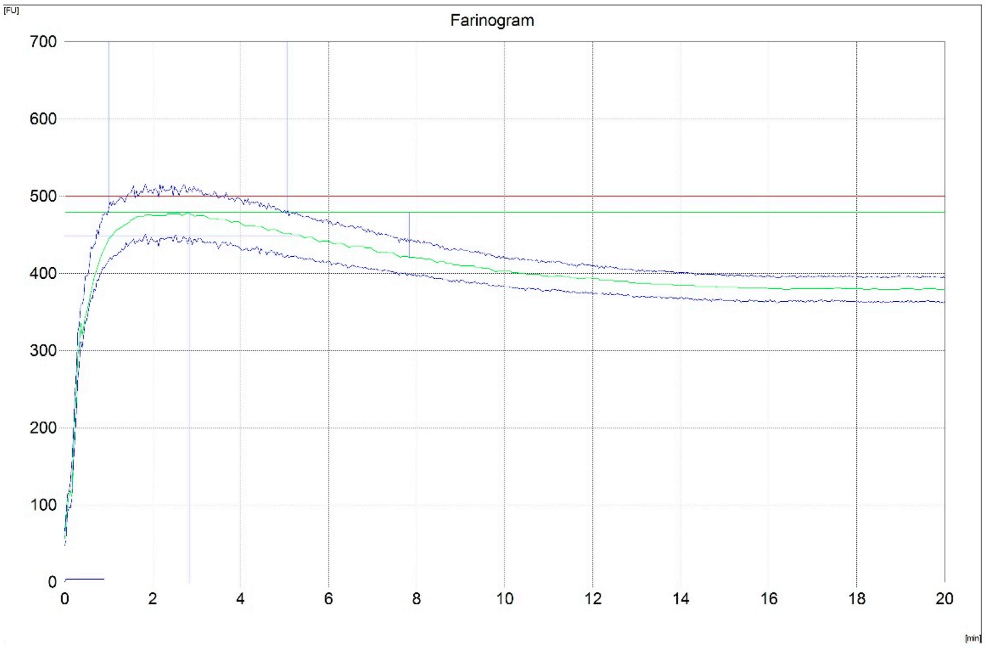

Test: C:\Users\cwbrabender\Documents\End Users 2024\T3 Rep 1.FAD

Brabender® Farinograph

Sample: T3 Rep 2
Date: 7/2/2024 6:45:34 PM

Method: AACC
Operator: TENG

Mixer: 50 g   Speed: 63 1/min
Consistency 502 FU with waterabsorption 57.3 %

Moisture content: 15.8 %

Waterabsorption (corrected for 500 FU):	57.3	%
Waterabsorption (corrected to 14.0 %):	59.4	%
Development time:	2.3	min
Stability:	3.9	min
Toleranceindex (MTI):	59	FU
Time to breakdown:	5.0	min
Farinograph quality number:	50	
Remarks:		

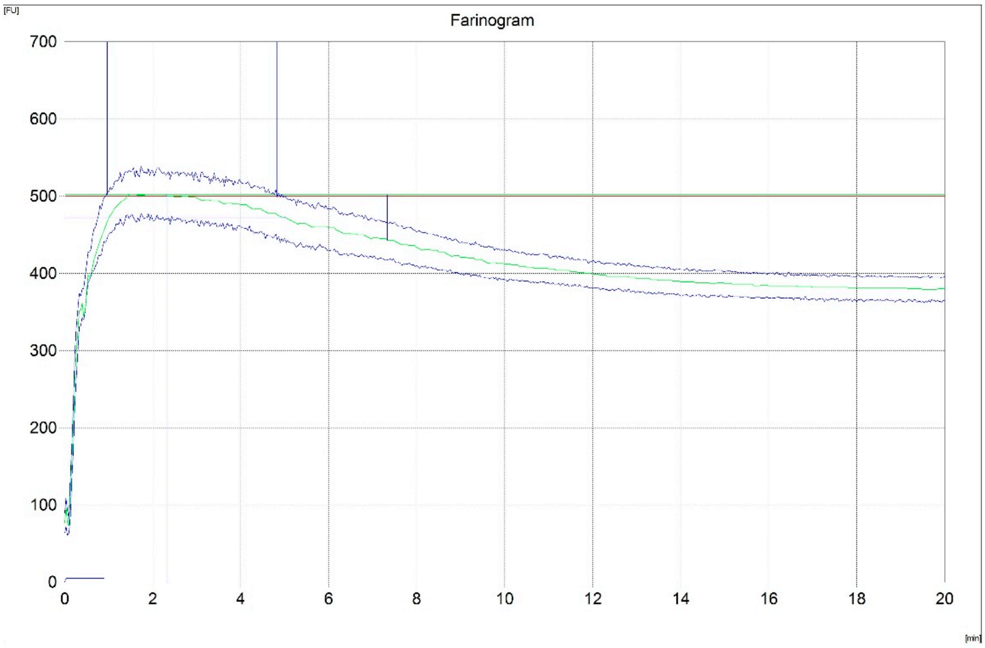

Test: C:\Users\cwbrabender\Documents\End Users 2024\T3 Rep 2.FAD

Brabender® Farinograph

Sample: T4 Rep 1		Method: AACC	
Date: 7/1/2024 10:40:55 PM		Operator: TENG	
Mixer: 50 g Speed: 63 1/min		Moisture content: 15.7 %	
Consistency 502 FU with waterabsorption 58.0 %			

Waterabsorption (corrected for 500 FU):	58.1	%
Waterabsorption (corrected to 14.0 %):	60.1	%
Development time:	1.7	min
Stability:	1.8	min
Toleranceindex (MTI):	79	FU
Time to breakdown:	3.0	min
Farinograph quality number:	30	
Remarks:		

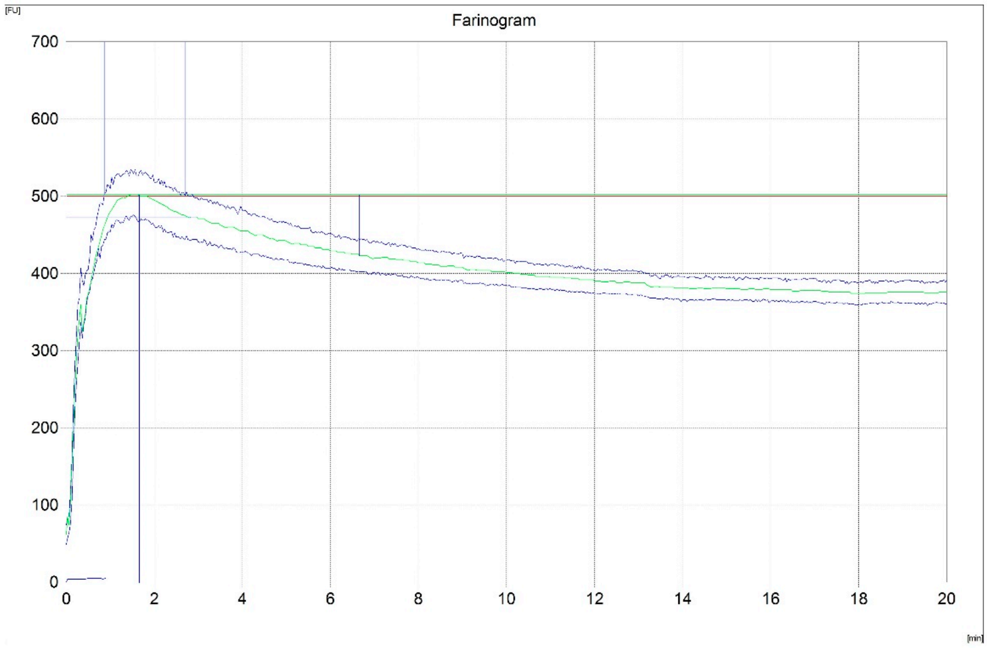

Test: C:\Users\cwbrabender\Documents\End Users 2024\T4 Rep 1.FAD

Brabender® Farinograph

Sample: T4 Rep 2	Method: AACC
Date: 7/2/2024 7:19:17 PM	Operator: TENG
Mixer: 50 g Speed: 63 1/min	Moisture content: 15.7 %
Consistency 502 FU with waterabsorption 58.1 %	

Waterabsorption (corrected for 500 FU):	58.1	%
Waterabsorption (corrected to 14.0 %):	60.1	%
Development time:	1.8	min
Stability:	2.0	min
Toleranceindex (MTI):	77	FU
Time to breakdown:	3.2	min
Farinograph quality number:	32	
Remarks:		

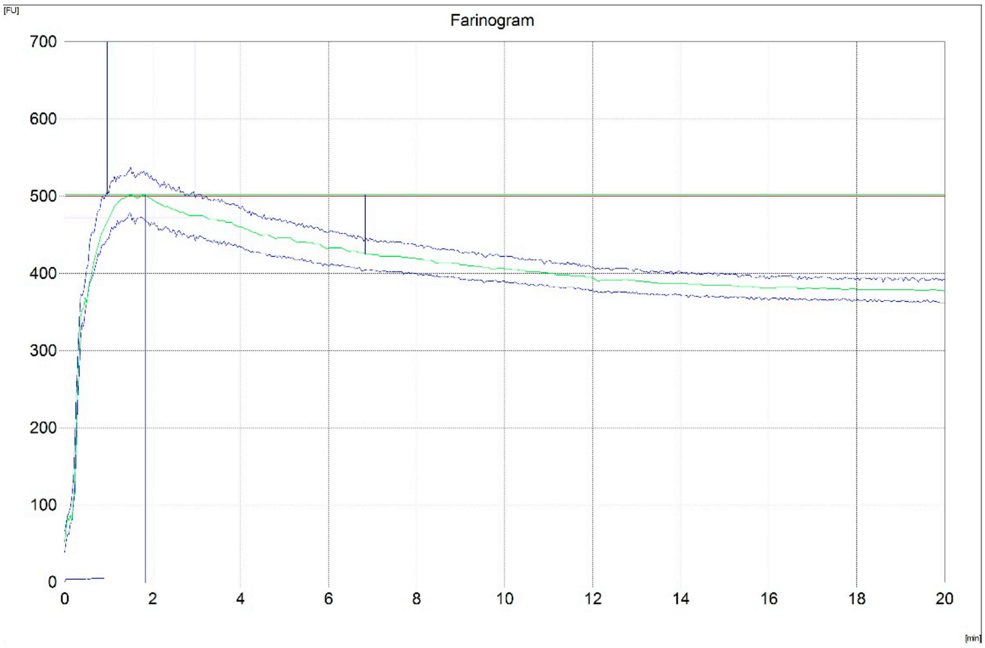

Test: C:\Users\cwbrabender\Documents\End Users 2024\T4 Rep 2.FAD

Brabender® Farinograph

Sample: T5 Rep 1
Date: 7/1/2024 11:33:02 PM

Method: AACC
Operator: TENG

Mixer: 50 g   Speed: 63 1/min
Consistency 509 FU with waterabsorption 60.2 %

Moisture content: 15.6 %

Waterabsorption (corrected for 500 FU):	60.4	%
Waterabsorption (corrected to 14.0 %):	62.3	%
Development time:	1.7	min
Stability:	1.8	min
Toleranceindex (MTI):	87	FU
Time to breakdown:	2.9	min
Farinograph quality number:	29	

Remarks:

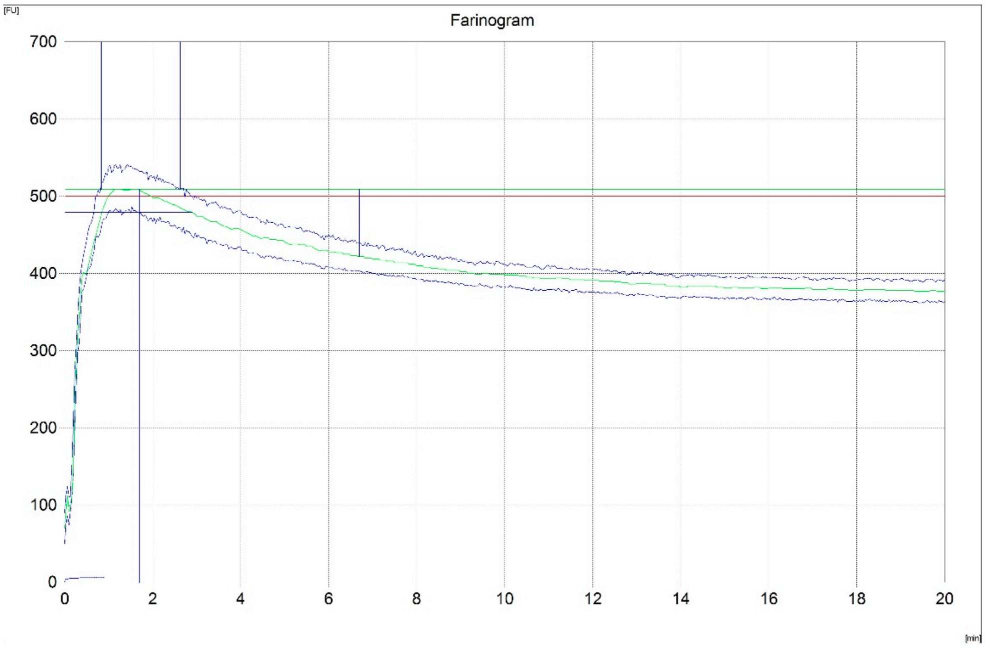

Test: C:\Users\cwbrabender\Documents\End Users 2024\T5 Rep 1.FAD

Brabender® Farinograph

Sample: T5 Rep 2
Date: 7/2/2024 7:42:06 PM

Method: AACC
Operator: TENG

Mixer: 50 g Speed: 63 1/min
Consistency 504 FU with waterabsorption 60.5 %

Moisture content: 15.6 %

Waterabsorption (corrected for 500 FU):	60.6	%
Waterabsorption (corrected to 14.0 %):	62.5	%
Development time:	1.7	min
Stability:	1.8	min
Toleranceindex (MTI):	88	FU
Time to breakdown:	2.8	min
Farinograph quality number:	28	

Remarks:

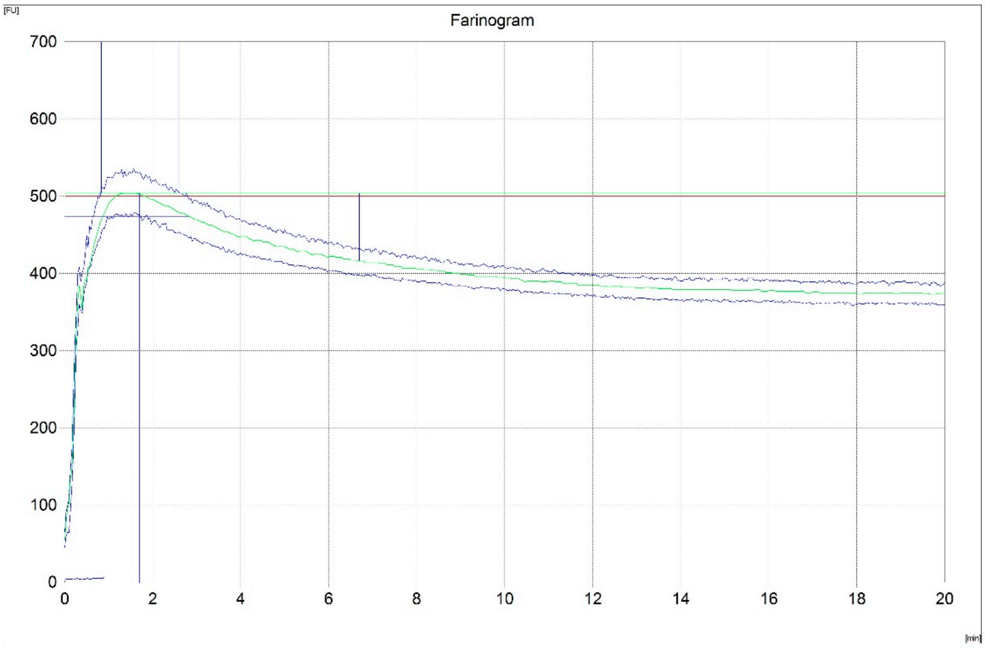

Test: C:\Users\cwbrabender\Documents\End Users 2024\T5 Rep 2.FAD
