# Supplementary figures and images for: Impact of Ferulated Arabinoxylans from Maize Bran on Farinograph and Pasting Properties of Wheat Flour Blends
Source: Foods. 2024 Oct 26;13(21):3414. doi: 10.3390/foods13213414 (PMC11545305; doi:10.3390/foods13213414)

## Supplementary File 2

Figure 1; Mixograms of T0 to T5 (duplicate samples)

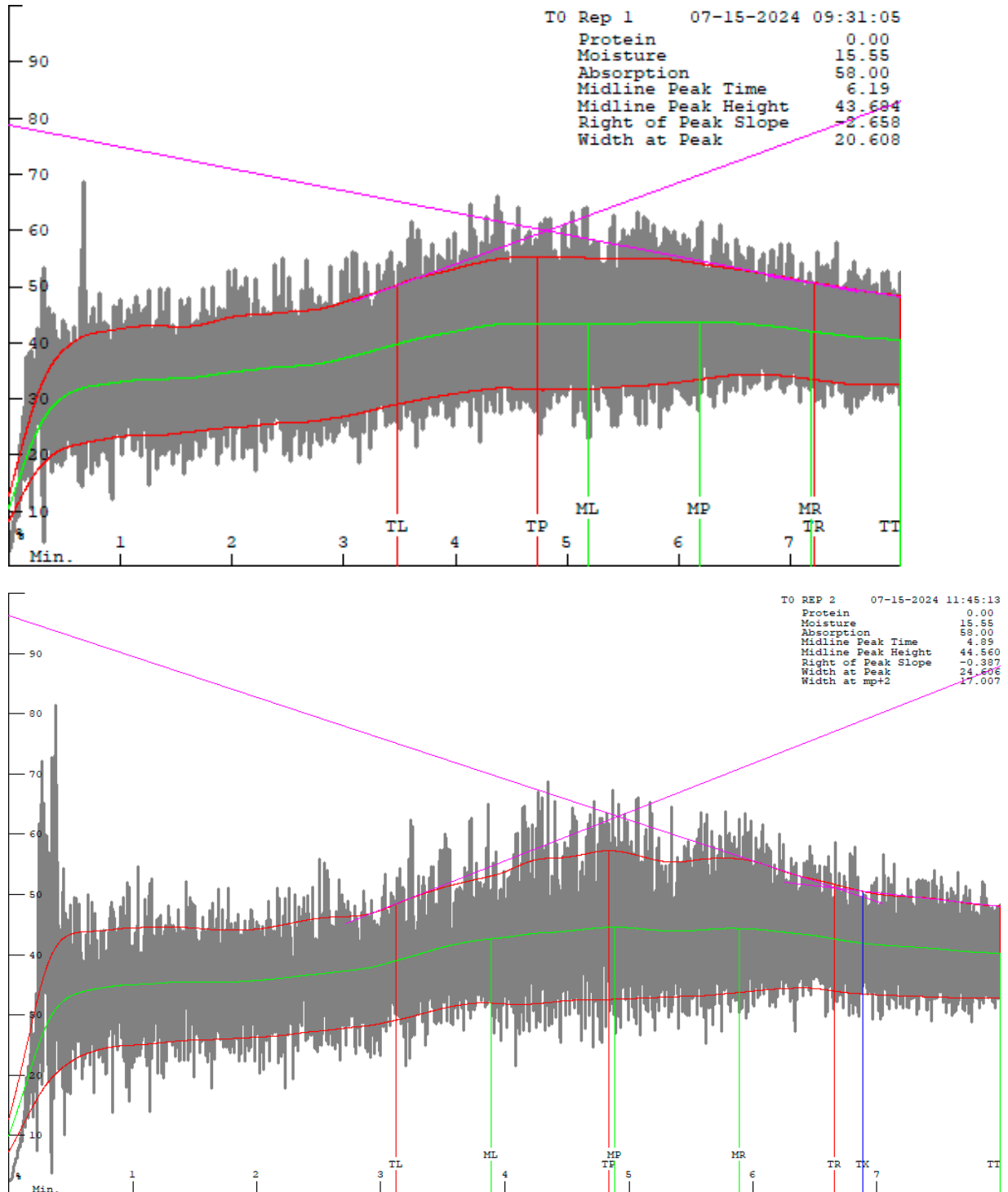

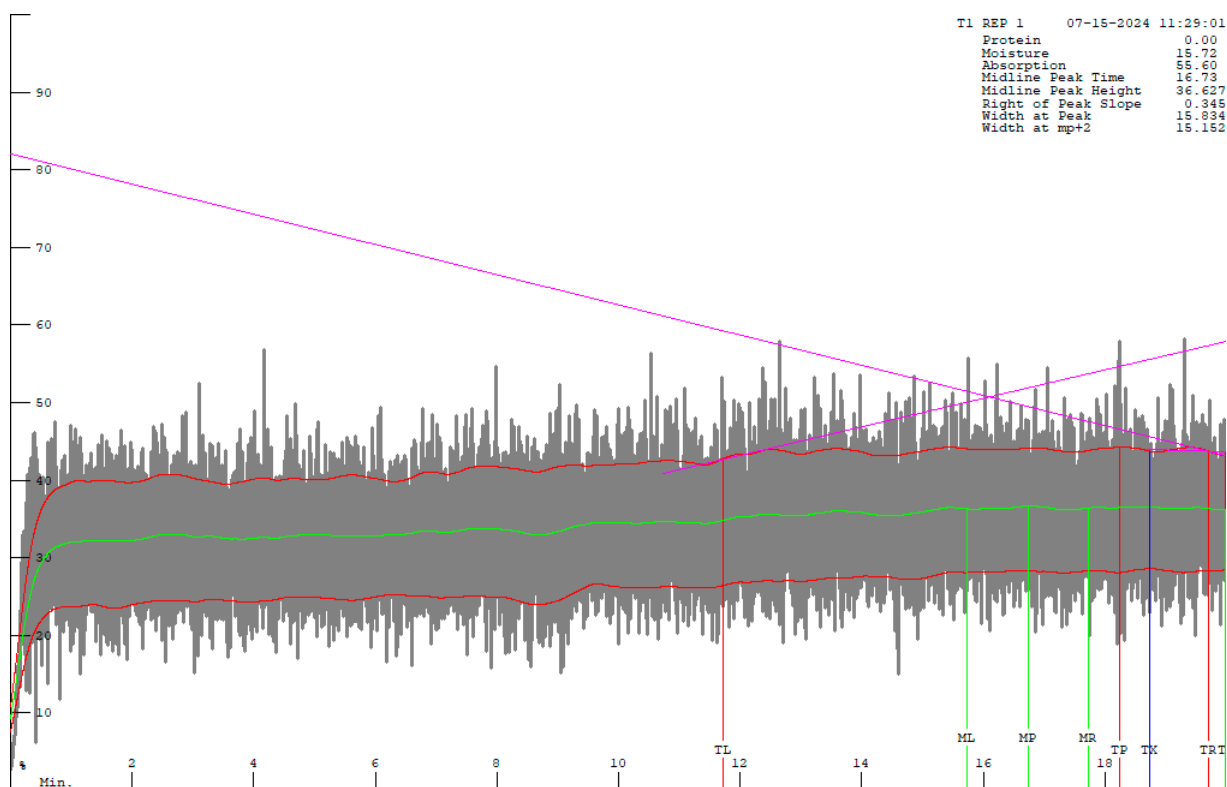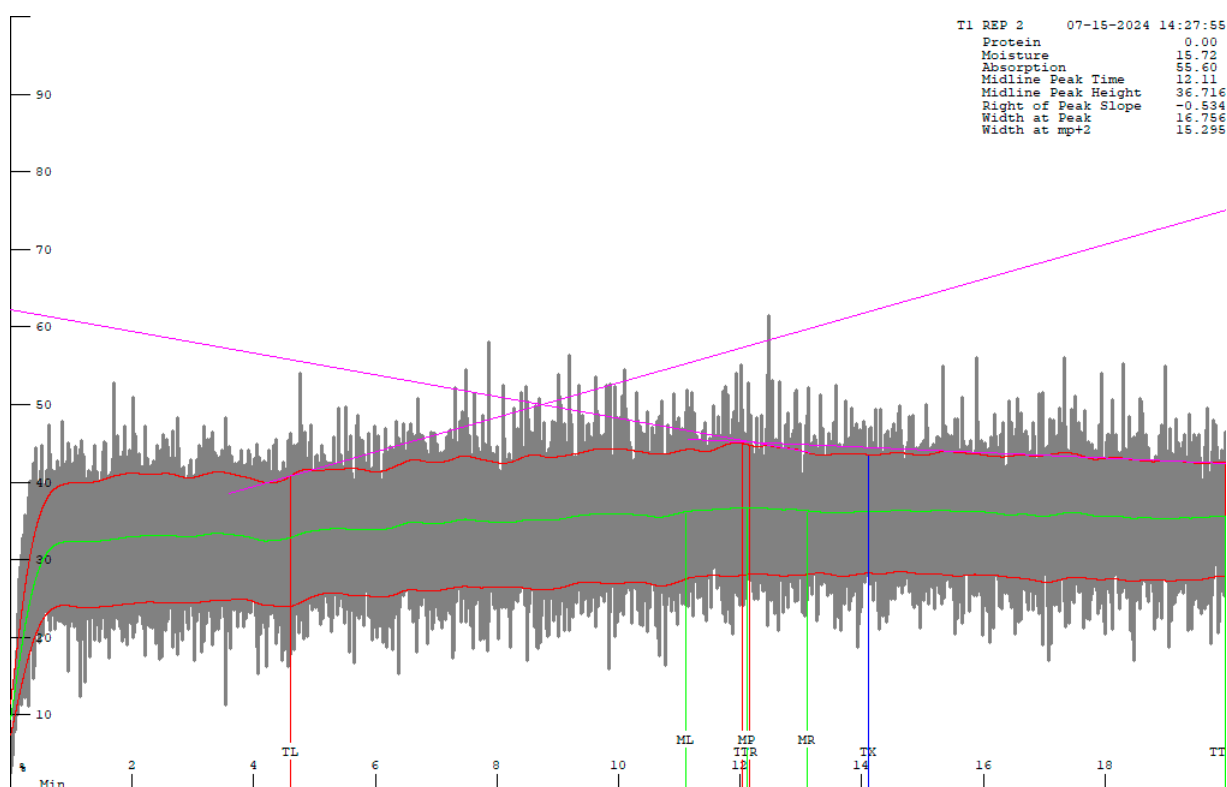

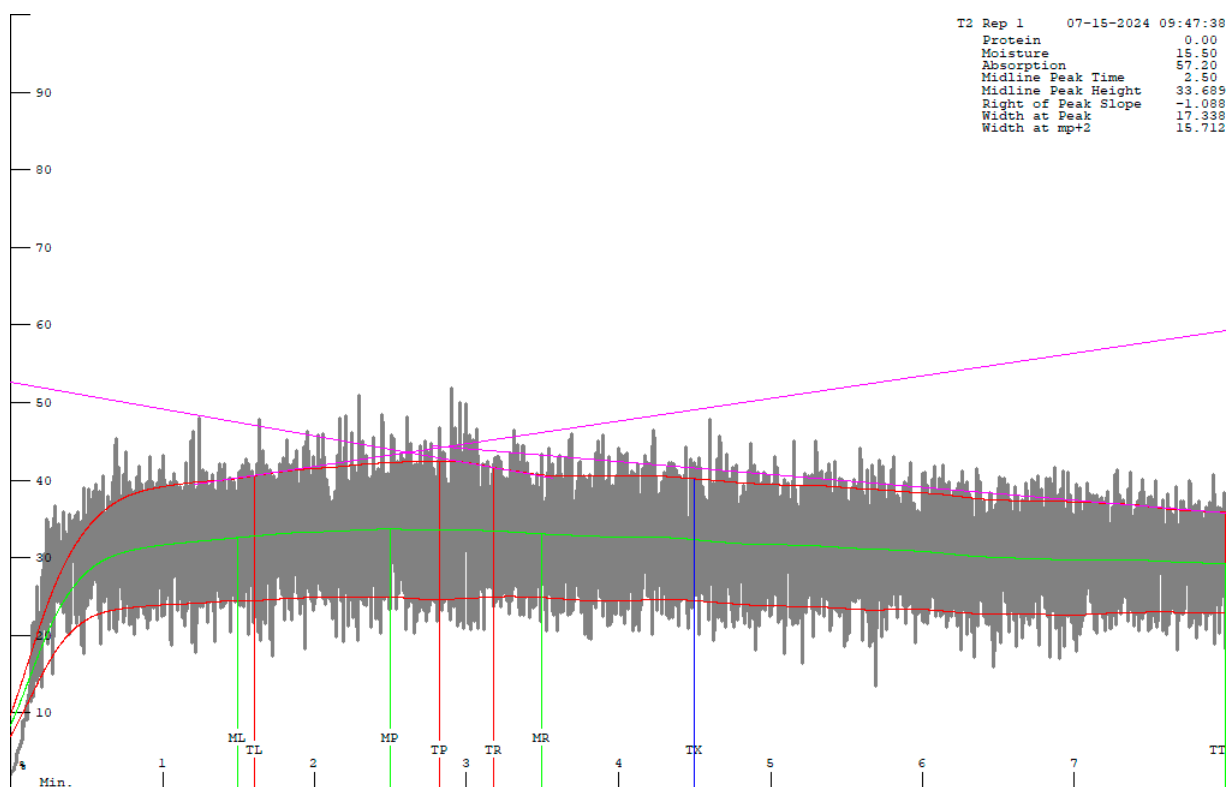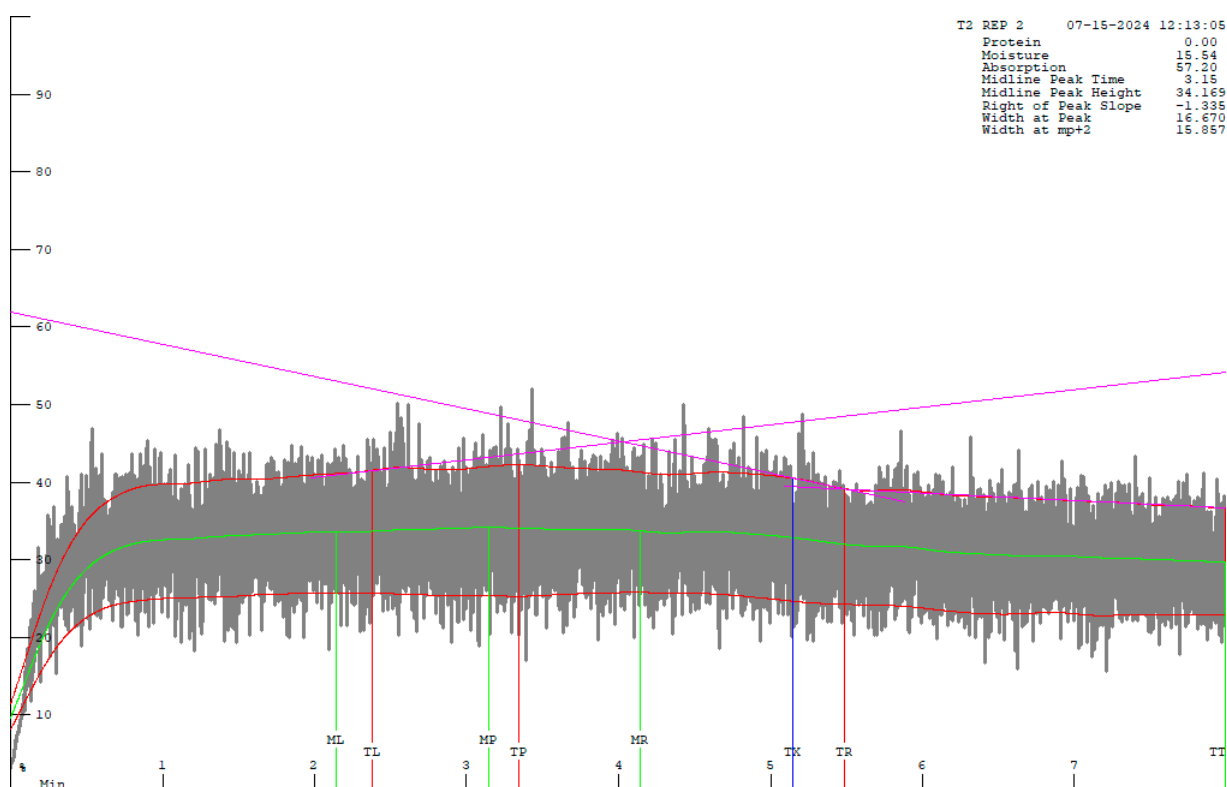

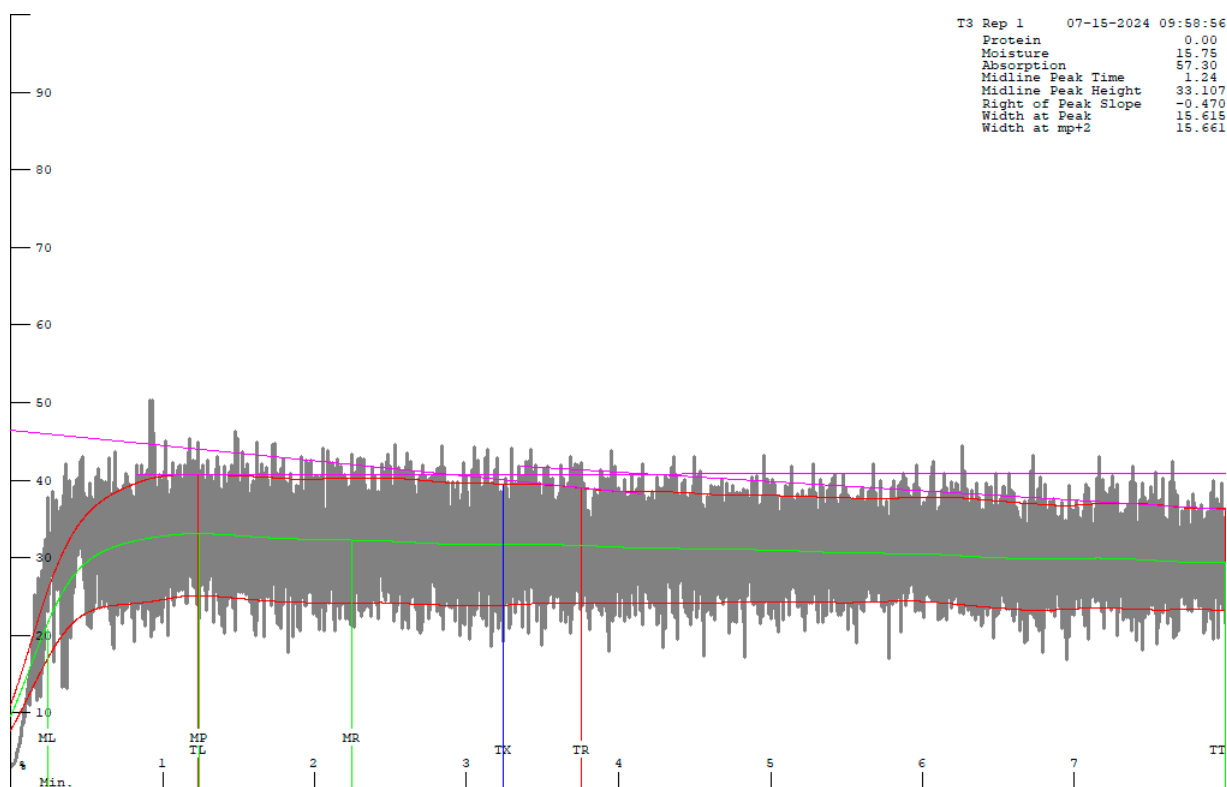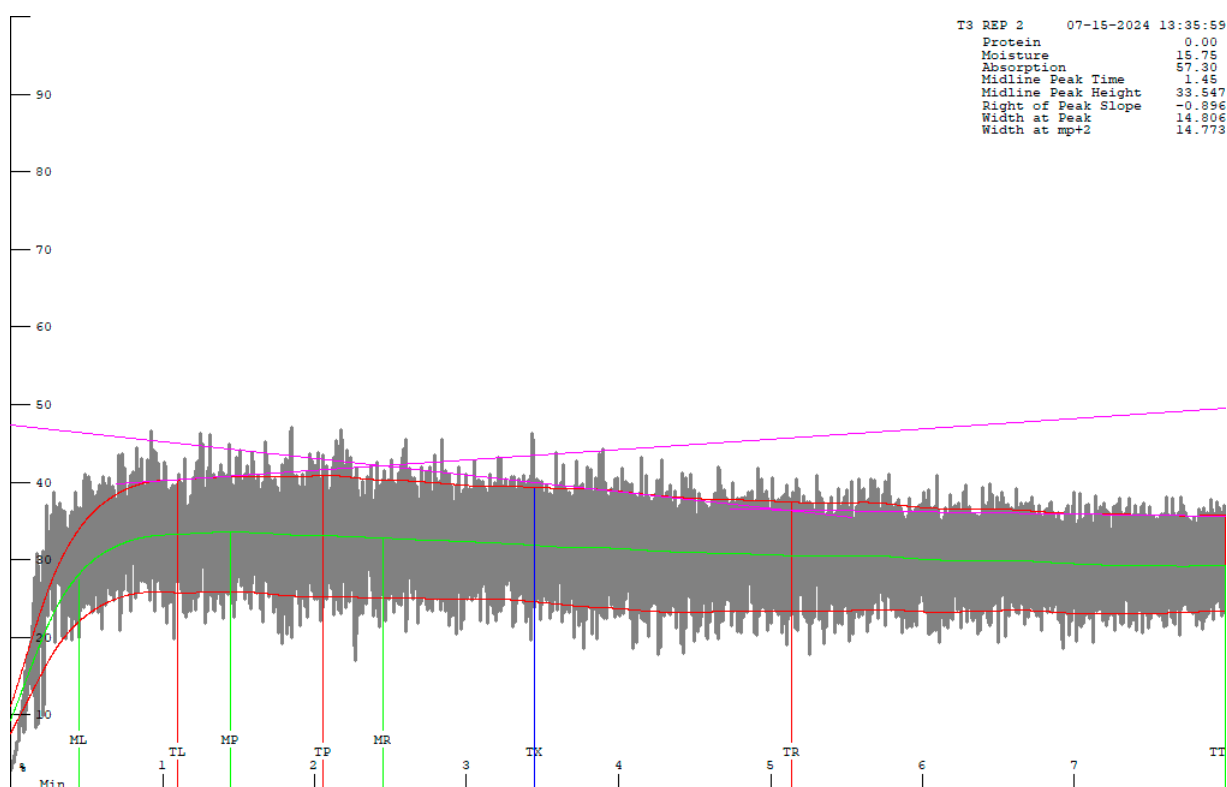

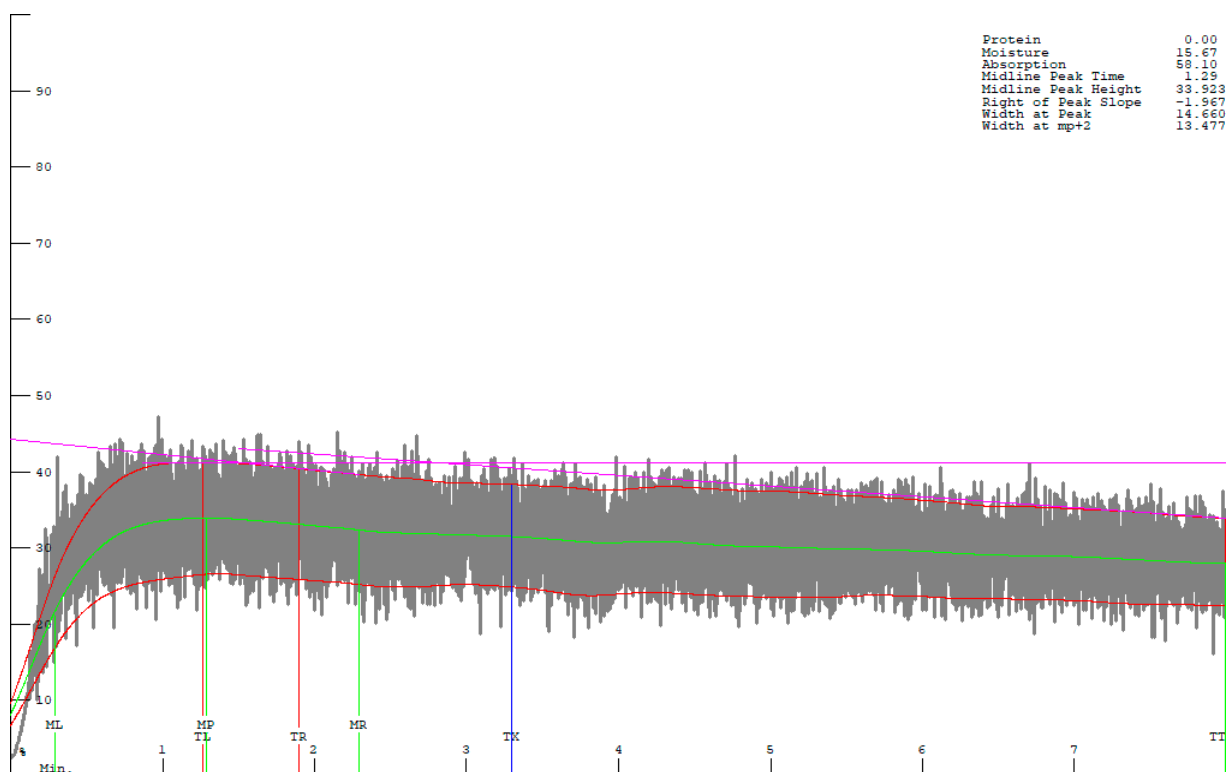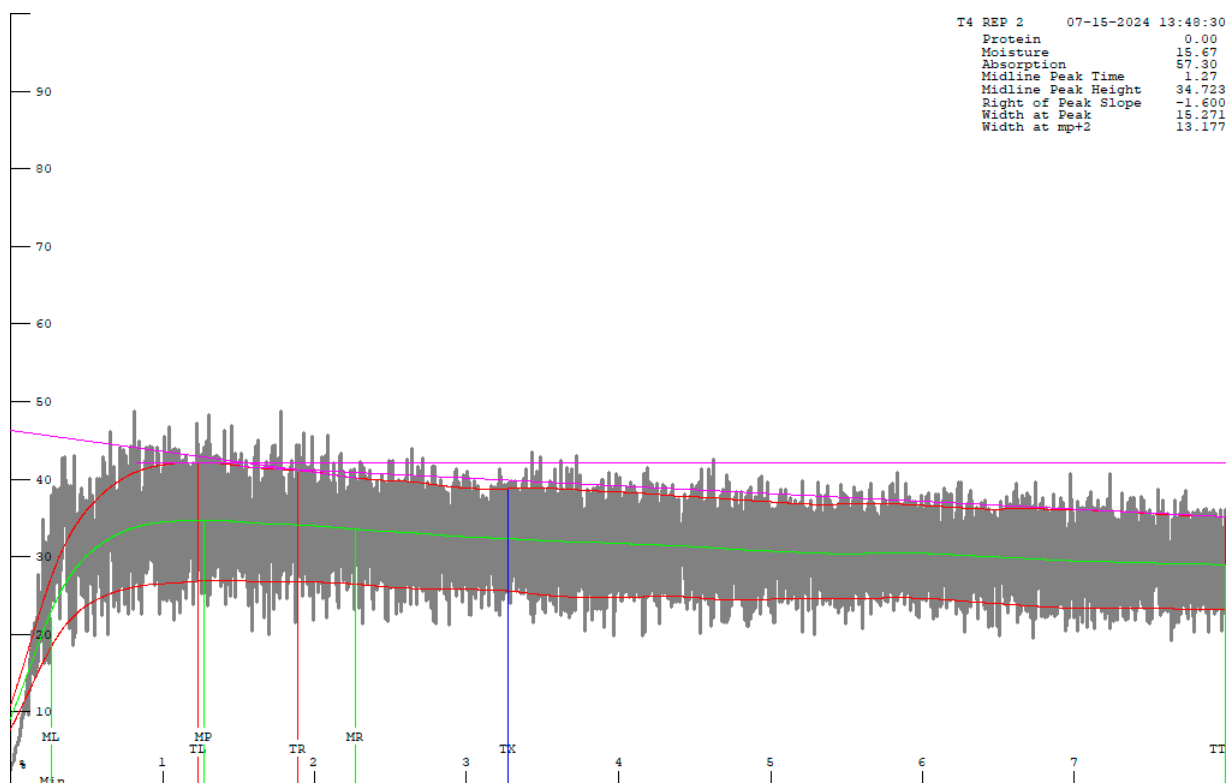

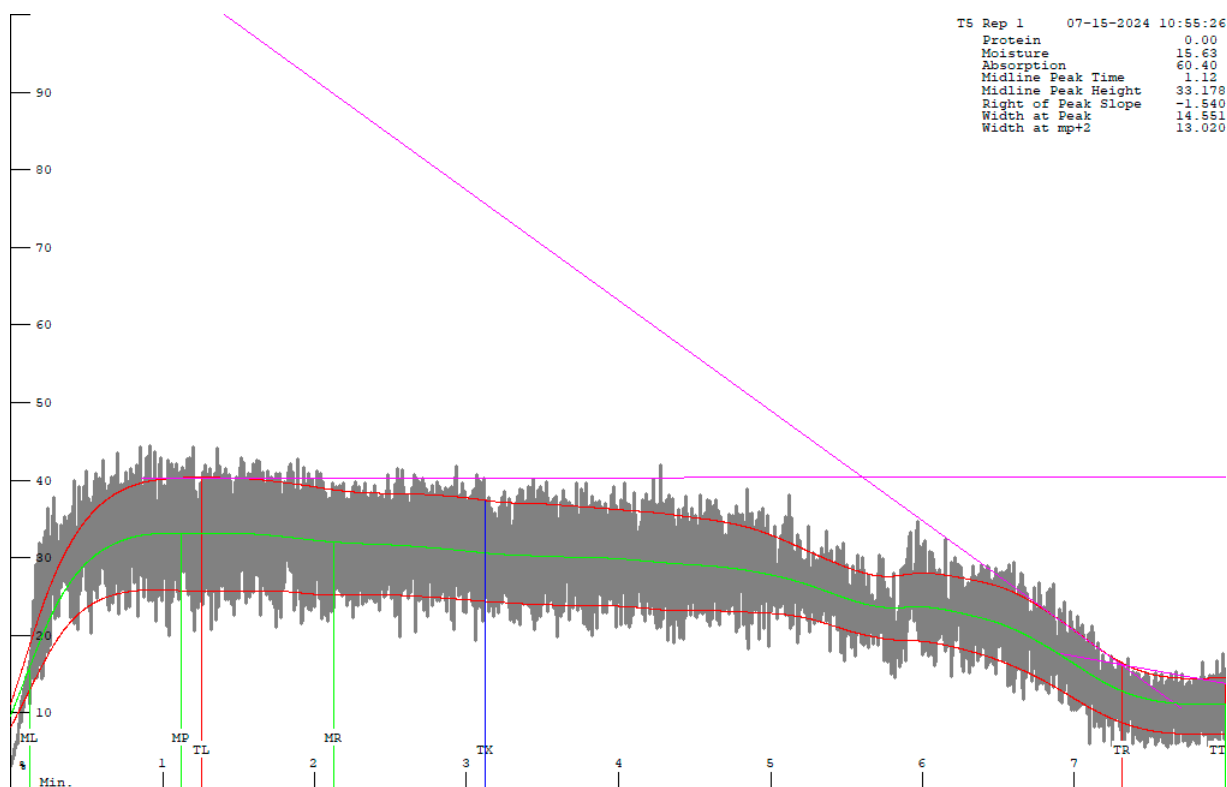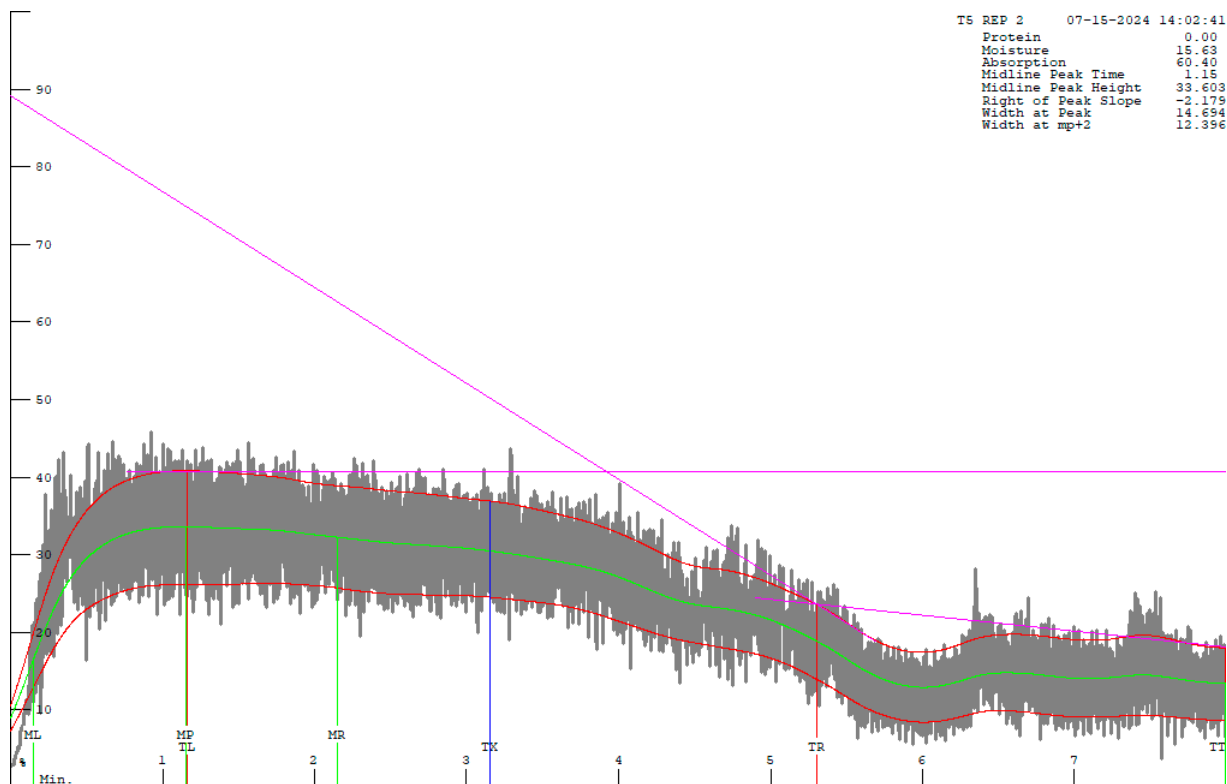

Supplement: Supplementary file 1 [file foods-13-03414-s001.zip › Supplementary File 2.pdf]
